# Immune Cell–Mediated Causal Link Between Gut Microbiota Traits and Childhood Asthma: Evidence From Mendelian Randomization and Metagenomic Sequencing

**DOI:** 10.1111/crj.70227

**Published:** 2026-08-23

**Authors:** Rui Shi, Zongming Yang, Xiuyun Zhou, Dong Xu, Wankai Xue, Lanfen An, Xiaole Zhang, Yongjian Huang

**Affiliations:** ^1^ Department of Pediatrics Tongji Hospital, Tongji Medical College, Huazhong University of Science and Technology Wuhan China; ^2^ Department of Plastic Surgery the First Affiliated Hospital of Anhui Medical University Hefei China; ^3^ Department of Nursing Tongji Hospital, Tongji Medical College, Huazhong University of Science and Technology Wuhan Hubei China

**Keywords:** childhood asthma, gut microbiota, immune cell, metagenomic sequencing, randomization study

## Abstract

**Background:**

The gut microbiota may be involved in childhood asthma. However, the causal relationship between the gut microbiota and childhood asthma remains obscure. Whether immune cells mediate the pathway from gut microbiota to childhood asthma has not been elucidated.

**Methods:**

Genetic data of 196 gut microbiota taxa, 731 immune cell phenotypes, and childhood asthma were retrieved from the MiBioGen consortium and the MRC‐IEU OpenGWAS database. Bidirectional Mendelian randomization (MR) analysis was first performed to verify the causal association between gut microbiota and childhood asthma, with reverse MR analysis conducted to rule out reverse causality. Mediation analysis was subsequently applied to identify the immune cell–mediated regulatory pathways. Additionally, a clinical validation cohort including childhood asthma patients and healthy controls was enrolled. Fecal samples from all subjects were subjected to metagenomic sequencing for microbial species identification and functional annotation. Linear discriminant analysis effect size (LEfSe) was used to screen differential gut microbiota taxa, whereas alpha diversity analysis was performed to evaluate microbial community richness. Kyoto Encyclopedia of Genes and Genomes (KEGG) analysis was utilized for microbial functional pathway annotation, and Python‐based bioinformatics analysis was adopted to quantify the abundance of microbial virulence factors based on Virulence Factor Database (VFDB) annotation results.

**Results:**

MR analysis confirmed significant causal associations between seven gut microbiota taxa and childhood asthma, among which four taxa exhibited robust causal effects, and no reverse causal relationship was detected. A total of 25 immune cell types showed statistically significant effects on childhood asthma risk. Mediation analysis further validated two key immune cell–mediated pathways: the CD64 phenotype on CD14–CD16 immune cells mediated the causal effect of *s_Paraprevotella_unclassified* on childhood asthma, and the CD45 phenotype on HLA‐DR T cells mediated the association of *s_Bacteroides_thetaiotaomicron* with childhood asthma. Clinical metagenomic sequencing revealed distinct gut microbial signatures between the two groups: The asthma group was characterized by enriched *Bacteroides*, whereas healthy controls had predominant *Akkermansia* and *Lachnospiraceae*, which was consistent with the MR findings. Alpha diversity analysis showed a trend of higher microbial species abundance in children with asthma without statistical significance. KEGG functional analysis indicated that differential microbial pathways between groups were primarily enriched in glucose metabolism, genetic information processing, and immune regulation. Moreover, the abundance of virulence factors including mrkl, mrkJ, mrkA, mrkB, mrkC, mrkD, mrkF, mrkH, impF, and hcp/tssD was significantly elevated in the childhood asthma group.

**Conclusions:**

Specific gut microbiota taxa exert definitive causal effects on childhood asthma. Two immune cell phenotypes serve as crucial intermediate mediators linking gut microbiota dysbiosis to childhood asthma development. These novel findings elucidate the microbiota‐immune regulatory mechanism underlying childhood asthma, providing a solid theoretical basis for the development of gut microbiota–targeted intervention strategies for childhood asthma prevention and treatment.

## Graphical abstract

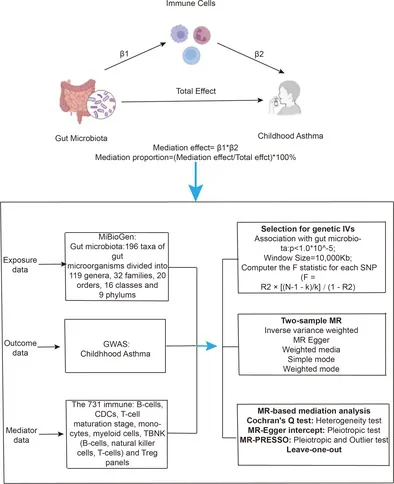

**Abbreviations**
COPD: chronic obstructive pulmonary disease
DC: dendritic cells
GWAS: genome‐wide association study
IV: instrumental variable
KEGG: Kyoto Encyclopedia of Genes and Genomes
LEfSe: linear discriminant analysis effect size
MR: Mendelian randomization
SCFA: short‐chain fatty acids
SNPs: single nucleotide polymorphisms
Tregs: regulatory T lymphocytes

## 1 Background

Asthma is a serious global health problem affecting people of all ages, which affects around 334 million people worldwide. The prevalence of asthma is increasing in many countries, especially among children [1, 2, 3]. Asthma is a common, complex, and heterogeneous disease that usually begins at an early age and is characterized by reversible airflow obstruction and clinical manifestations of recurrent episodes of coughing, wheezing, and dyspnea [4]. By definition, the early stages of asthma, bronchiolitis, or early wheezing affect 10%–30% of children, and as many as 15%–25% of children experience recurrent wheezing before school age [5]. Eighteen asthma‐associated genomic loci were identified by the genome‐wide association studies (GWAS), including five novel loci at 5q31.3, 6p22.1, 6q15, 12q13.3, and 17q21.33. The 22 different genome‐wide significant single nucleotide polymorphisms (SNPs) corresponding to the 18 loci explained 3.5% of the variation in asthma susceptibility [6]. Many risk factors have been shown to directly or indirectly influence the development and progression of asthma, including genetic and environmental factors such as allergens, airway pollution, and viral infections. Childhood asthma displays unique metabolomic profiles suggesting specific underlying mechanisms, such as modulation of host‐pathogen and gut microbiota interactions, epigenetic changes in T‐cell differentiation, and low antioxidant properties of the airway epithelium [7]. The underlying causes of asthma are not completely understood. Although the prevalence of asthma has been controlled by various methods, the prevalence is currently at high levels. Therefore, it is of paramount importance to study asthma in children.

As the human gut microbiota is a highly complex system, the gut microbiota plays a pivotal role in human health through its interactions with the host [8, 9, 10]. Recent studies have provided evidence for a potential bidirectional gut–airway axis. The information exchange between the gut and the respiratory tract regulates microbial–immune interactions that have a major impact on the clinical diagnosis and treatment of a range of respiratory diseases, including asthma, chronic obstructive pulmonary disease (COPD), and lung cancer [11, 12, 13]. The increasing amount of research demonstrated a relationship between the gut microbiome and asthma. For example, antibiotic use and alterations in the composition of the gut microbiome are associated with the risk and severity of asthma [14, 15, 16, 17, 18]. The mechanisms by which gut microbiota influences asthma pathogenesis are unknown. Gut microbiota could modulate the host immune system through metabolites (e.g., short‐chain fatty acids [SCFA] and lipopolysaccharides). For example, specific flora (e.g., the genus *Clostridium*) promotes regulatory T cell (Treg) differentiation and suppress Th2‐type inflammatory responses, thereby reducing asthma risk [16, 19]. In addition, several cross‐sectional studies have shown an association between the gut microbiota and asthma. For example, Demirci et al. found reduced mucinophilic *Ackermannia* and *E. pulae* in a group of children with asthma compared with a group of healthy children. A Danish prospective cohort study on asthma showed that higher abundance of *Veillonella* and lower abundance of *Roseburia*, *Alistipes*, and *Flavonifractor* at 1 year of age were associated with an increased risk of asthma at 5 years of age [20, 21]. Although studies have shown an association between gut microbiota and asthma, these studies are limited by factors such as participant selection bias, confounders, and reverse causality. Therefore, the mechanism of interaction between gut microbiota and asthma remains uncertain.

Gut microbiota mediates immune cells to influence the development of multiple diseases. Importantly, the gut microbiota plays a regulatory role in lung immune function and inflammatory responses. Emerging evidence indicates that symbiotic gut microbiota undergoes continuous cell division and death, releasing microbial components, metabolic by‐products, and extracellular vesicles. These bioactive substances can modulate host immune function and further affect the progression of respiratory diseases [22]. For example, gut microbiota could influence the onset and progression of ARDS by triggering inflammatory responses, immune cell dysfunction, oxidative stress, apoptosis, autophagy, pyroptosis, and ferroptosis. Through T‐cell receptor signaling, SCFA activates IL‐22, regulatory T cells, and Th2 cells in the lungs, thereby reducing inflammation and reducing the incidence of lung cancer. B cells, T cells, and other immune cells can be activated by the gut microbiota to infiltrate the lungs via the blood or lymphatic pathways. The activation triggers a pulmonary immune response that leads to episodes of lung diseases such as COPD and asthma [23, 24, 25]. However, studies of gut microbiota mediating the effects of immune cells on asthma, especially childhood asthma, are scarce, and the molecular mechanisms are uncertain. Therefore, it is essential to study the causal relationship between gut microbiota, immune cells, and childhood asthma to provide a better understanding of the pathogenesis of asthma and to guide gut microbiota–orientated interventions in clinical practice.

Mendelian randomization (MR) is a statistical method that uses genetic variation as an instrumental variable (IV) to estimate the causal effect of exposure factors on outcomes in the presence of unmeasured confounders [26]. With the tremendous increase in genetic data on microbiota and complex diseases, MR has been widely employed in recent years. The purpose of this study was to investigate the causal role of gut microbiota on childhood asthma and whether it could be mediated by immune cells. Initially, we collected SNP data as an IV for exposure. Then, a comprehensive two‐sample MR analysis was conducted to assess the causal role of gut microbial and immune cell profiles on childhood asthma. Subsequently, we investigated the effects of gut microbiota on immune cell profiles and calculated the proportion of immune cell profile‐mediated effects of gut microbiota on childhood asthma in order to assess whether gut microbiota can influence the progression of childhood asthma through regulation of the immune system. Ultimately, fecal samples of the subjects were collected, and species identification and functional annotation of the microbiota were completed via metagenomic sequencing. The sequencing data further corroborated the findings derived from our prior MR analysis.

## 2 Methods

### 2.1 Study Design

**FIGURE 1:**
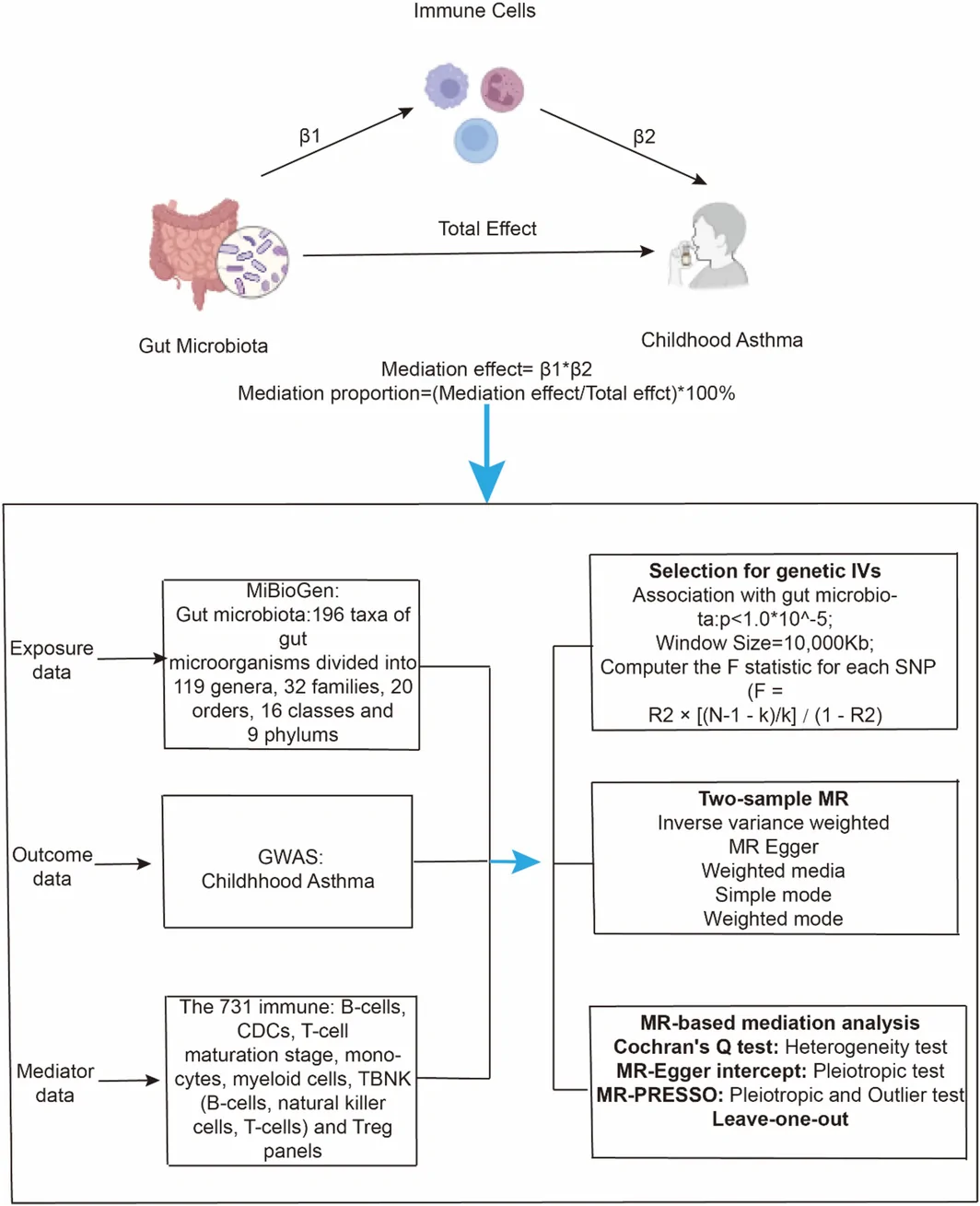
Flowchart of the MR study. A two‐step Mendelian randomization study of gut microbiota on childhood asthma mediated by immune cell.

A two‐step MR was performed to determine the relationship between gut microbiota and asthma risk in children and whether immune cell characteristics could mediate this association. This study consisted of three main components, as shown in Figure 1: investigating the causal effect of gut microbiota on childhood asthma; analyzing the causal effect of immune cells on childhood asthma; and implementing mediation analysis to examine the role of gut microbiota–mediated immune cells in the pathway to childhood asthma. SNPs were defined as IVs [27].

### 2.2 Data Sources

Gut microbiota data of the study were obtained from the MiBioGen consortium (https://mibiogen.gcc.rug.nl/), including 196 taxa of gut microorganisms divided into 119 genera, 32 families, 20 orders, 16 classes, and 9 phyla. The extensive research was coordinated using 16S rRNA gene sequencing profiles on 18 340 individuals from 24 cohorts with consistent European ancestry, ensuring population homogeneity and avoiding ancestral stratification bias [28].

Orru et al. [29] published pooled GWAS data for 731 immune cell traits in a dataset comprising 3757 individuals of European ancestry with no overlapping cohorts. The 731 immune cell characteristics can be categorized into six groups: B cells, CDCs, T‐cell maturation stage, monocytes, myeloid cells, TBNK (B cells, natural killer cells, and T cells), and Treg panels. In addition, the 731 immunophenotypes included median fluorescence intensity (*n* = 389), absolute cell count (AC) (*n* = 118), relative cell count (*n* = 192), and morphological parameters (*n* = 32).

The childhood asthma GWAS dataset used in this study was derived from the IEU OpenGWAS database with the official accession ID ukb‐d‐ASTHMA_CHILD. This dataset included a total of 314 633 European participants, consisting of 13 962 childhood‐onset asthma cases and 300 671 healthy controls. The phenotype was strictly defined as doctor‐diagnosed asthma with disease onset before 19 years of age. All cases were diagnosed following standardized pediatric respiratory clinical criteria, whereas individuals with COPD, bronchiectasis, and other confounding respiratory diseases were excluded.

### 2.3 IV Selection

We used the “TwoSampleMR” package to set the linkage disequilibrium (LD) threshold to *R* ^2^ < 0.001 with an aggregation window size of 10 000 kb in 1000 Genomes EUR data to identify IVs. The *F* statistic was calculated according to the given formula: *F* = *R* ^2^ × [(N − 1 − k)/k]/(1 − *R* ^2^), where *K* represents the number of genetic variants, *N* represents the size of the sample, and *R* ^2^ represents the total variance accounted for by the selected SNPs. Ultimately, we calculated the *F*‐statistic for each SNP to detect any weak IV bias in our analyses. SNPs with *F*‐statistics lower than 10 indicate a possible weak IV bias. After obtaining GM exposure data from public databases, we identified 3279 gut microbiota–associated SNPs and 22 480 immune cell–associated SNPs as IVs after excluding weak IVs associated with gut microbiota and immune cells by setting the significance threshold to *p* < 1 × 10^−5^, and the *F*‐statistics ranged from 20 to 60.93 (Table S1).

### 2.4 Statistical Analysis

To assess the potential causal relationship between gut microbiota, immune cells, and childhood asthma, an independent two‐sample MR analysis was performed. As sensitivity analyses, up to five MR methods were used to generate effect estimates, which make differing pleiotropy assumptions. The inverse variance weighted (IVW) is a method of calculating an analytically weighted average of random variables that uses the variance of each random variable as a weight thus helping to reduce the volatility of the mean [30]. The IVW method combined with Cochran's *Q* test, which was employed to detect heterogeneity of SNPs, where *p* < 0.05 indicates the presence of heterogeneity [31], was adopted to estimate the overall causal effect. The MR‐Egger intercept test and MR‐PRESSO global test were used to identify horizontal pleiotropy. Scatter plots and leave‐one‐out plots were generated for visual inspection. Bidirectional MR was additionally performed to rule out the possibility of reverse causality. All analyses were performed in R 4.3.1 software using the “two‐sample MR,” “gwasglue” and “VariantAnnotation” packages for data processing and results visualization.

Two‐step mediation analysis is a method of decomposing the direct effect of exposure (gut microbiota) on an outcome (childhood asthma) as well as the effect through mediating variables. First, we analyzed the causal effect between exposure and outcome using SNP effect. Subsequently, we evaluated the causal effects of intermediate biomarkers significantly driven by exposure on the outcome. The overall effect can be decomposed into an indirect effect (via the mediating variable) and a direct effect (no mediating variable) [32]. The overall effect of gut microbiota on childhood asthma can be decomposed into (1) a direct effect on childhood asthma (c) and (2) an indirect effect of gut microbiota via the mediating variable (a × b). We calculated the percentage of the mediating effect by dividing the indirect effect by the total effect.

### 2.5 Patient Cohorts

Thirty‐three participants were enrolled in our study, including 25 patients diagnosed with childhood asthma from Tongji Hospital, Tongji Medical College, Huazhong University of Science and Technology, and 8 age‐matched healthy controls. All pediatric asthma participants enrolled in this study were recruited from Tongji Hospital, Tongji Medical College, Huazhong University of Science and Technology, between 2025 and 2026. All patients were clinically diagnosed with childhood asthma in strict accordance with the Chinese Guidelines for the Diagnosis and Prevention of Childhood Asthma (2025 Edition), with an age inclusion criterion of over 6 years and no gender restrictions. Additionally, all enrolled patients had no history of antibiotic or probiotic use within 1 week prior to sample collection and were free of acute respiratory tract infections during the enrollment period. Age‐matched healthy children were recruited as the control group from the same medical center, who met identical exclusion criteria, including no antibiotic or probiotic administration and no active respiratory infection within 1 week before enrollment. Prior to fecal sample collection, the relevant procedures were informed to the children and their families, and written informed consent was obtained; the study protocol was approved by the Ethics Committee of Tongji Hospital. All clinical fecal samples involved in this study were prospectively collected strictly after the acquisition of ethical approval. Exclusion criteria for all participants included digestive system diseases, autoimmune diseases, and pulmonary infectious diseases. Participants had not been administered antibiotics, antifungals, probiotics, inhaled corticosteroid, or prebiotics for at least 2 months before sampling. However, we recruited age‐matched children with newly diagnosed allergic asthma without chronic inhaled corticosteroid use, yet BMI, sex, feeding patterns, pet contact, secondhand smoke exposure, geographic location, and asthma severity were not controlled. Fresh fecal samples were collected, transported to our laboratory in an ice bag within 2 h, and then stored at −80°C until testing.

### 2.6 DNA Extraction and Metagenome Sequencing

The fecal samples were processed for DNA extraction (using the QIAamp Fecal Pro DNA Kit), quality control, and DNA library construction (with a DNA concentration > 3 nM). Metagenomic shotgun sequencing was performed on the Illumina HiSeq platform.

### 2.7 Metagenomic Analysis of Stool Samples and Bioinformatic Analysis

Quality‐controlled or host‐depleted sequencing data were taxonomically annotated using Kraken2. Annotation outputs were further statistically analyzed and formatted via Bracken and KrakenTools, whereas species distribution was visualized with Krona. Species diversity was assessed using customized Python and R scripts. LEfSe was applied to screen differential microbial biomarkers across groups, and two‐tailed *t*‐tests were performed to identify species with significantly altered abundance. For functional annotation, clean reads were assembled using MEGAHIT or SPAdes, followed by genomic sorting and statistical analysis in BioTool. Assembled contigs were annotated with Kraken2, and non‐microbial sequences were excluded to retain microbial genomes. Gene prediction was conducted using MetaGeneMark, and nonredundant gene and protein sets were generated via CD‐HIT. Protein sequences were functionally annotated against TIGRFAMs, Pfam, and GO databases using InterProScan and aligned to the KEGG database with DIAMOND. Only alignment hits with coverage > 30% were reserved for final functional profiling.

## 3 Results

### 3.1 Association Between Gut Microbiota and Childhood Asthma

**FIGURE 2:**
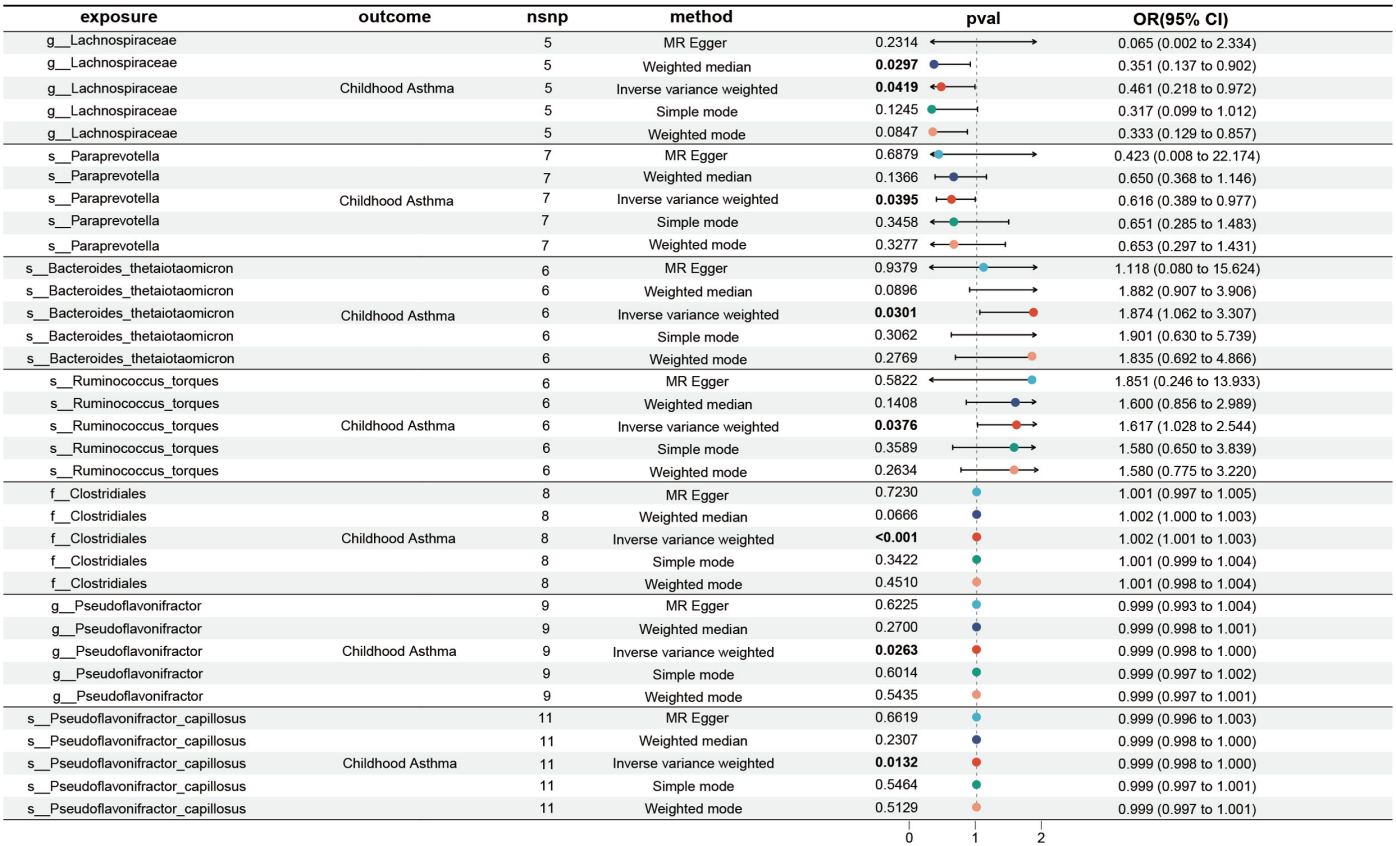
Mendelian randomization analysis between gut microbiota and childhood asthma. MR analysis showed the causality of eight genera on childhood asthma was significant (*Bacteroides_thetaiotaomicron*, *Ruminococcus_torques*, *Clostridiales*, *Lachnospiraceae*, *Paraprevotella*, *Pseudoflavonifractor*, and *Pseudoflavonifractor_capillosus*). CI, confidence interval; GM, gut microbiota; MR, Mendelian randomization; OR, odds ratio; SNP, single nucleotide polymorphism.

Childhood asthma was associated with seven different types of gut microbiota, divided into three phyla, four families, five genera, and four species. For more information on the 52 SNPs for these seven gut microbiota types, see Table S2. IVW approach with a significance threshold of *p* < 0.05 was used as a filter to screen for GMs associated with childhood asthma disease. Results indicated that three gut microbiotas were positively associated with the development of childhood asthma: *s_Bacteroides_thetaiotaomicron* (OR = 1.874, 95% CI = 1.062–3.307, *p* < 0.0301), *s_Ruminococcus_torques* (OR = 1.617, 95% CI = 1.028–2.544, *p* = 0.0376), and *f_Clostridiales* (OR = 1.002, 95% CI = 1.000–1.003, *p* < 0.001). The four gut microbiota that were negatively associated with childhood asthma included *g_Lachnospiraceae* (OR = 0.461, 95% CI = 0.218–0.972, *p* = 0.0419), *s_Paraprevotella* (OR = 0.616, 95% CI = 0.389–0.977, *p* = 0.0395), *g_Pseudoflavonifractor* (OR = 0.999, 95% CI = 0.998–1.000, *p* = 0.0263), and *s_Pseudoflavonifractor_capillosus* (OR = 0.999, 95% CI = 0.998–1.000, *p* = 0.0132) (Figure 2). Furthermore, the stability of the primary results was largely confirmed by these analyses through heterogeneity, multiplicity, and sensitivity tests (Figure S1 and Figure S2). Moreover, the reverse MR analysis yielded no statistically significant results (Table S3). Finally, Steiger filtering was performed to verify the causal direction, exclude SNPs with reverse causal effects, and improve the reliability of MR results (Table S4).

### 3.2 Immune Cell and Childhood Asthma

**FIGURE 3:**
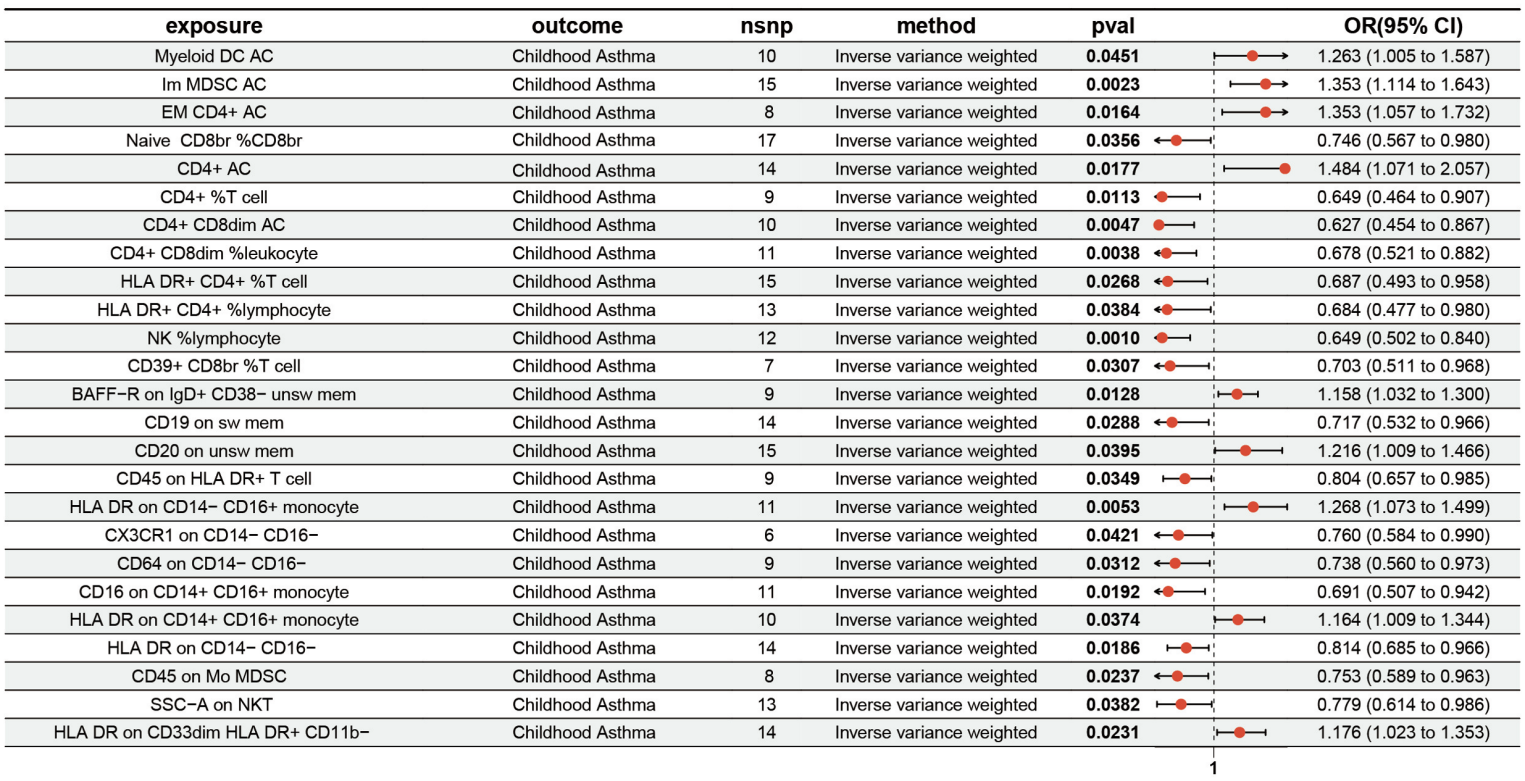
Forest plot illustrating the causal effects between immune cell phenotypes and childhood asthma as determined by MR analysis. MR analysis investigated the association between 25 immune cell types and childhood asthma. IVW, inverse‐variance‐weighted; OR, odds ratio; SNP, single nucleotide polymorphism.

We initially selected 731 immunophenotypes to analyze the effect on childhood asthma (Table S5). The study of the relationship between these immunophenotypes and childhood asthma revealed several important associations. MR analysis investigated the association between 25 immune cell types and childhood asthma. Results indicated that nine immune cells were negatively associated with the development of childhood asthma, including myeloid cells, monocytes, and immune‐activated cells. Conversely, one‐sixteen immune cells were positively associated with the development of childhood asthma, incorporating T cells, B cells, NK cells, monocytes, and MDSC (Figure 3). Among these, T‐cell lineages exerted the predominant effect, consistent with previous published evidence. Furthermore, the stability of the primary results was largely confirmed by these analyses through heterogeneity and multiplicity tests (Table S6). Leave‐one‐out sensitivity analyses further confirmed the robustness of causal effect estimates. Sequential exclusion of each instrumental SNP individually yielded nearly identical overall effect sizes (Figure S3 and Figure S4).

### 3.3 Effects of Gut Microbiota on Immune Cells

**FIGURE 4:**
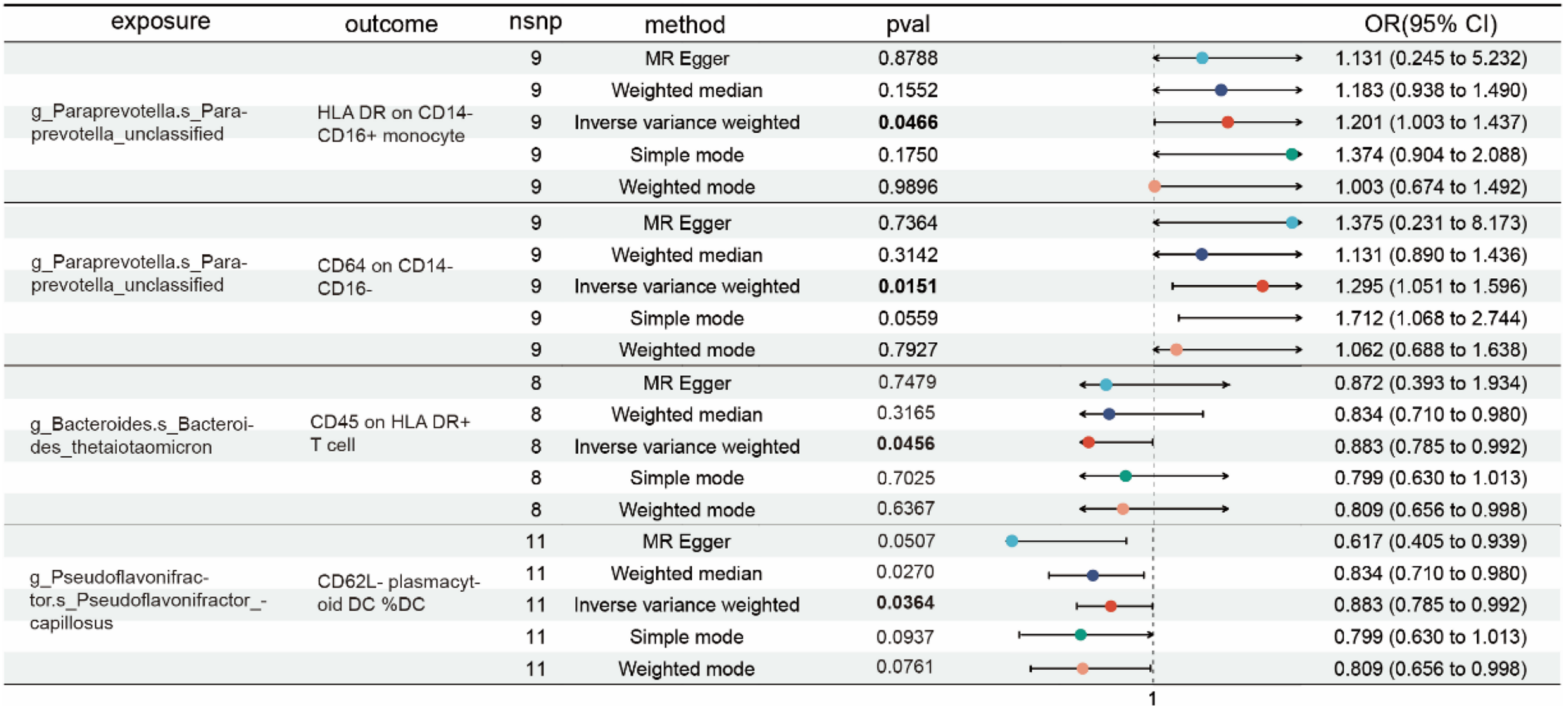
MR analysis between gut microbiota and immune cells. MR analysis showed 16 immunophenotypes had protective effects on childhood asthma and 9 immune cell traits had disadvantageous effects on childhood asthma. OR, odds ratio; SNP, single nucleotide polymorphism.

Based on the results of the previous section, 25 immunophenotypes were identified as potential mediators. Subsequently, a comprehensive MR analysis was performed to investigate the influence of the gut microbiota on these mediators. We found that genus *Paraprevotella* could increase the level of HLA DR on CD14–CD16^+^ monocyte (OR = 1.202, *p* = 0.0466) and CD64 on CD14–CD16^−^ (OR = 1.295, *p* = 0.0151). However, genus *Pseudoflavonifractor* decreased the level of CD62L‐plasmacytoid DC% DC (OR = 0.883, *p* = 0.0364). Besides, genus *Bacteroides* could decrease the level of CD45 on HLA DR^+^ T cell (OR = 0.883, *p* = 0.0456) (Figure 4). Sensitivity analyses including heterogeneity, multiplicity and sensitivity tests indicated no evidence of directional pleiotropy (Figure S5).

### 3.4 Exploration of the Mediator of the Gut Microbiota and Childhood Asthma

**FIGURE 5:**
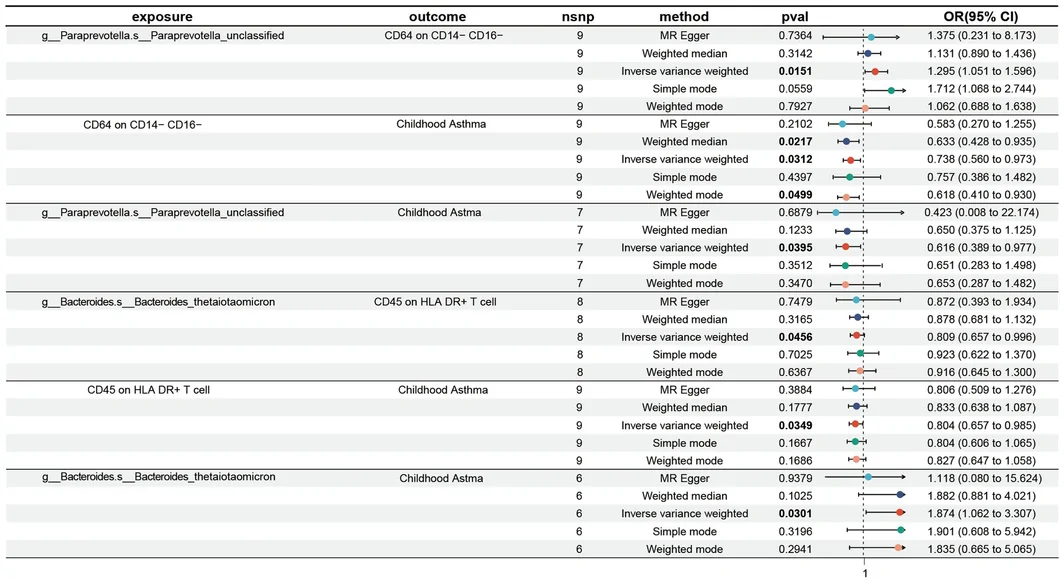
Causal relationships between gut microbiota and childhood asthma mediated by immune cells. Causal relationship between *Paraprevotella* and childhood asthma and the negative regulation of CD64 on CD14–CD16^−^ cells. Causal relationship between *Bacteroides_thetaiotaomicron* and childhood asthma and the positive regulation of CD45 on HLA DR^+^ T cells.

In identifying the key mediators influencing childhood asthma and the effects of gut microbiota on these mediators, a mediation analysis was performed. Specifically, we first assessed the effect of gut microbiota on immune cell phenotypes (β1) and then examined the role of immune cell phenotypes in mediating the development of childhood asthma using the same approach (β2). Ultimately, we identified two immune cell phenotypes that play a significant role in mediating the relationship between gut microbiota and childhood asthma. The results showed that the effects of *s_Paraprevotella* and *s_Bacteroides_thetaiotaomicron* on the risk of asthma development in children were mediated by immune cells CD64 on CD14–CD16^−^ and CD45 on HLA DR^+^ T cells, where the mediating proportions were 16.2% and 7.35% (Table S6). When CD64 on CD14–CD16^−^ cells acted as a mediator between *s_Paraprevotella* and childhood asthma, we found that the gut microbiota was associated with an increase in CD64 on CD14–CD16^−^ cells, which in turn was negatively associated with an elevated risk of childhood asthma. Likewise, when CD45 on HLA DR^+^ T cells were used as an intermediate medium, *s_Bacteroides_thetaiotaomicron* was associated with a decrease in CD45 on HLA DR^+^ T cells, which in turn was positively associated with childhood asthma risk (Figure 5).

Altogether, these findings emphasize the complex mediating role of the immune cell phenotype in the relationship between gut microbiota and childhood asthma, providing new insights into the underlying mechanisms of childhood asthma development.

### 3.5 Gut Microbiota Characteristics in Asthmatic Children and Healthy Children

**FIGURE 6:**
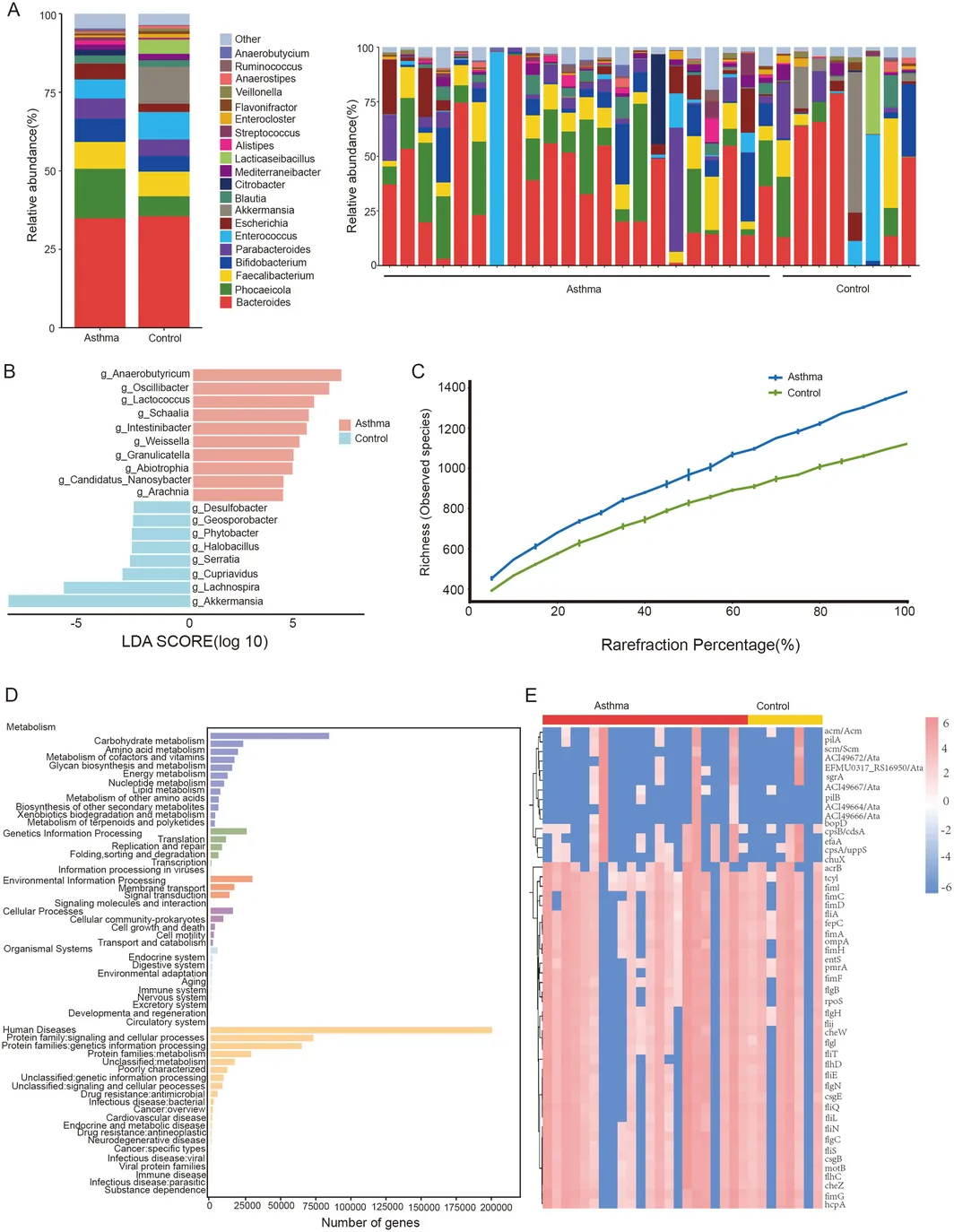
Gut microbiota characteristics in asthmatic children and healthy children. (A,B) Read‐based species abundance bar plot of fecal samples (asthmatic vs. healthy children): Top 20 species by maximum relative abundance. (C) Rarefaction curve of alpha diversity for read‐based species annotation. (D) KEGG‐based functional annotation of nonredundant protein‐coding sequences. (E) VFDB‐based virulence factor abundance table (asthma vs. healthy group, Python‐collated): Top 50 factors by average abundance for heatmap generation.

Metagenomic sequencing revealed significant differences in the composition of the gut microbiome between the childhood asthma group and the healthy control group. The characteristic microbiota of the childhood asthma group was identified as the genus *Bacteroides*, whereas those of the healthy control group were identified as the genera *Akkermansia* and *Lachnospiraceae* (Figure 6A,B). Our metagenomic sequencing results partially corroborate the outputs of the previous MR analysis. Alpha diversity analysis indicated that the species abundance within the pediatric asthma group was higher than that in the healthy control group, with no statistically significant difference observed (Figure 6C). This result indicated only a trend without statistical significance. Based on the KEGG annotation of nonredundant gene sets, Python was used to generate an abundance table reflecting the gene abundance of each sample across diverse pathways. LEfSe analysis was subsequently performed to identify differential pathways. KEGG functional analysis demonstrated that the differential microbial functional pathways between childhood asthma group and the healthy control group were associated with glucose metabolism, as well as various processes and mechanisms related to genetic information. The asthma group was predominantly enriched in pathways including oxidative phosphorylation, one‐carbon pool by folate, base excision repair, lipoic acid metabolism, and cell cycle—*Caulobacter*. Among these, oxidative phosphorylation, one‐carbon pool by folate, lipoic acid metabolism, mineral absorption, and carotenoid biosynthesis are closely associated with immune regulation (Figure 6D and Figure S6A). However, the observed correlation is only preliminary bioinformatic prediction without metabolomic validation, and causal linkage cannot be confirmed. Based on the VFDB database, we collated the virulence factor abundance table for the childhood asthma group and the healthy group using Python and selected the top 50 virulence factors by average abundance to generate an abundance heatmap. The results indicated that the childhood asthma group had higher abundances of the virulence factors mrkl, mrkJ, mrkA, mrkB, mrkC, mrkD, mrkF, mrkH, impF, and hcp/tssD (Figure 6E and Figure S6B,C). These virulence‐related factors are closely associated with *Klebsiella pneumoniae* , *Klebsiella oxytoca* , *Pseudomonas aeruginosa* , and other bacterial strains. Metagenomic sequencing results indicated that the gut microbiota characteristics of the pediatric asthma group differed from those of the healthy control group. These results confirm from distinct perspectives that the gut microbiota plays a crucial role in the onset and progression of childhood asthma.

## 4 Discussion

Asthma, a chronic inflammatory disease of the airways, is a prevalent chronic childhood disease with a heavy global health burden. The complex etiology and pathogenesis of asthma involves both genetic and environmental factors, posing challenges for diagnosis, severity prediction, and treatment strategies [33]. Imbalance of gut microbiota is strongly associated with many childhood diseases, such as autism, growth retardation, functional gastrointestinal disorders in children, inflammatory bowel disease, and asthma [34]. Previous studies have shown that the gut microbiota, that is, in the first year of life, is associated with asthma and may represent a critical window in early life with lasting effects on long‐term health in children [35]. By using two‐sample MR, we found an extremely significant causal effect of seven gut microbiotas on childhood asthma. Of these MiBioGen 16S‐based, *s_Bacteroides_thetaiotaomicron*, *s_Ruminococcus_torques*, and *f_Clostridiales* were positively associated with childhood asthma, suggesting that increased abundance of these gut microbiotas may increase the risk of childhood asthma. In addition, we identified a number of gut microbiotas that were protective against childhood asthma, including *g_Lachnospiraceae*, *s_Paraprevotella*, *g_Pseudoflavonifractor*, and *s_Pseudoflavonifractor_capillosus*. Metagenomic sequencing indicated that the characteristic microbiota of the asthmatic children group was identified as the genus *Bacteroides*, whereas those of the healthy control group were identified as the genera *Akkermansia* and *Lachnospiraceae*. The results of metagenomic sequencing analysis of the gut microbiota in children with asthma provide several valuable insights into the role of the gut microbiota in asthma disease. Studies have shown that the high‐cellulose dietary reduces asthma symptoms by altering the composition of the gut microbiota, for example, *s_Ruminococcus_torques*. However, this mechanism is thought to be independent of SCFA and may involve regulation of lipid metabolism [36]. Nevertheless, the microbial profiles of other intestinal segments remain poorly characterized in the context of childhood asthma, and the findings of the present study provide a theoretical foundation for future relevant research.

Recent studies have elucidated the complex relationship between asthma and factors such as immune regulation, genetic susceptibility, viral infections, and allergen exposure, which may collectively lead to immune dysregulation that induces asthma. Immune cells, particularly dendritic cells (DCs) and regulatory T lymphocytes (Tregs), play a key role in childhood asthma attacks [37]. For example, sputum from asthmatic children has a markedly reduced population of T regulatory cells, suggesting impaired immune tolerance in asthmatic children. Elevated levels of peripheral blood DC subpopulations in asthmatics emphasize the critical role of DCs in antigen presentation, T‐cell activation, and the development of asthma and allergic reactions [38]. Cytokine‐producing Th subsets including Th1 (IFN‐γ), Th2 (IL‐4, IL‐5, IL‐13), Th9 (IL‐9), Th17 (IL‐17), Th22 (IL‐22), and T regulatory (IL‐10) cells have all been suggested to play an important role in the pathogenesis of asthma and regulate the clinical manifestations and progression of asthma by driving airway inflammation, promoting eosinophil infiltration, and airway remodeling. It can regulate the clinical manifestations and progression of asthma by driving airway inflammation and promoting airway remodeling [39, 40]. However, the underlying mechanisms linking immune cells to the development of asthma are not clear. In our study, the relationship between these 25 immunophenotypes and childhood asthma revealed several important associations, including T cells, B cells, NK cells, monocytes, and MDSC. Twenty‐five immunophenotypes were determined as potential mediators. Comprehensive MR analysis was subsequently performed to investigate the influence of the gut microbiota on these mediators. *Paraprevotella* increased the levels of CD14–CD16^+^ monocyte and CD64 on CD14–CD16^−^ cell; however, *Pseudoflavonifractor* and *Bacteroides_thetaiotaomicron* reduced the levels of DC% DC and CD45 on HLA DR^+^ T cell. Next, a mediation analysis was performed to identify the key mediators influencing childhood asthma and the effects of gut microbiota on these mediators. *Paraprevotella* reduces the incidence of childhood asthma by an increase in the levels of CD64 on CD14−CD16^−^ cells. *Bacteroides_thetaiotaomicron*, in turn, was positively associated with childhood asthma risk through the mediating factor CD45 on HLA DR^+^ T cells. The research indicated that CD64^+^ CD14^−^CD16^−^ monocytes serve as potential contributors to age‐related macular degeneration (AMD), and their potential immunomodulatory crosstalk with retinal pigment epithelial (RPE) cells has been uncovered [41]. Moreover, researches focusing on CD45^+^HLA‐DR^+^ T cells in the context of respiratory diseases remain scarce. The study conducted by Chen et al. [42] demonstrated that activated T cells with high HLA‐DR expression exacerbate pulmonary injury via oxidative stress and cellular apoptosis. Peripheral CD4^+^ T cells from patients with isolated asthma exhibit no significant upregulation of HLA‐DR. In contrast, elevated levels of activated T cells are observed only in patients with COPD and asthma–COPD overlap (ACO). These findings indicate that HLA‐DR^+^ T cells serve as a specific immune biomarker for structural airway damage characteristic of COPD, with no remarkable alterations seen in patients with simple allergic asthma. However, the functional mechanisms of CD64‐expressing CD14^−^CD16^−^ cells and CD45‐expressing HLA‐DR^+^ T cells in childhood asthma remain unclear and warrant further investigation.

The gut microbiota actively interacts with the host immune system through the secretion of bacterial structural components and metabolites, thereby modulating the ability of the gastrointestinal tract to mount local and systemic immune responses and affecting various distal parts of the host, including the lungs. SCFA is the most abundant metabolite of the gut microbiota and is considered a critical mediator of bidirectional gut–lung interactions [43]. Studies have shown that oral administration of SCFA to mice protects against the development of asthma by weakening DCs to prevent Th2 cell activation and cytokine production by Th2 cells [44]. Bidirectional regulation of the gut–lung axis and host immunity can mediate gut dysbiosis and asthma. Research [45] has shown that *Paraprevotella* strains isolated from the fecal microbiome of healthy human donors are effective trypsin‐degrading symbiotic bacteria. Mechanistically, *Paraprevotella* promotes trypsin autolysis by recruiting trypsin to the bacterial surface via a type IX secretion system‐dependent polysaccharide anchoring protein. Trypsin‐degrading symbiotic colonization may contribute to the maintenance of intestinal homeostasis and protection against pathogen infection. In a study that included 40 bronchial asthmatics and 15 healthy individuals, the abundance of *Paraprevotella* was reduced in the features of bronchial asthma relative to healthy individuals [46]. Furthermore, *Paraprevotella* has been associated with a variety of diseases, such as colorectal cancer, coronary plaque characteristics, and Alzheimer's disease [47, 48, 49]. Previous studies have shown that *Bacteroides* are key bacterial genera in the early gut colonization process and are influenced by a variety of environmental factors such as infant diet, mode of delivery, and number of siblings and family members [50]. Changes in gut microbiota, such as differences in *Bacteroides_thetaiotaomicron*, observed in Black and White children in one study, and its association with a history of asthma in that column, suggest a potential role for *Bacteroides_thetaiotaomicron* in the higher prevalence of asthma observed in Black children with allergies [51]. In the MR analysis, the results indicate that *Paraprevotella* is negatively associated with childhood asthma; nevertheless, *Bacteroides_thetaiotaomicron* is the opposite, which is consistent with the above findings and provides an important basis for further childhood asthma research. Our study investigated the functional annotation and analysis of the gut microbiota in children with asthma. We compared the functions and related pathways of the gut microbiota between asthmatic children and healthy controls. Our analysis revealed that the glucose pathways were significantly enriched in the gut microbiota of children asthma group, as well as various processes and mechanisms related to genetic information. Previous studies have indicated a close association between obesity and the development and progression of childhood asthma. In the present study, KEGG enrichment analysis revealed a strong correlation with glucose metabolism, which will serve as an innovative focus for our future research.

Our study was distinguished by its comprehensive approach, integrating several rigorous analyses to delve into the association between gut microbiota and childhood asthma. A series of sensitivity analyses were used in this study to maximize the robustness of the MR results. The innovation of this study lies in the adoption of metagenomic sequencing technology, which has a more robust species annotation capability than amplicon technology. This technology was applied to identify the characteristics of the gut microbiota in asthmatic children, analyze the relevant functions of the microbiota, and compare the gut microbiota features among asthmatic children. These findings provide a theoretical reference for a more comprehensive understanding of the role of the gut microbiota in the occurrence and progression of childhood asthma. Research on the gut microbiota will offer novel targets and insights for the early prediction of childhood asthma, as well as for identifying therapeutic targets for intestinal mucosal immune abnormalities during its pathogenic process. Nevertheless, this study is subject to several limitations. The main limitation is that the data for the GWAS study were European, which may introduce some bias and limit the broad applicability of our findings to other ethnic groups. In addition, although we collected clinical data to validate the MR analysis by metagenomic sequencing, our study still has the following limitations: a small sample size, a wide age range, the inability to match the per‐meal food intake between the patient and healthy control groups, the lack of targeted metabolomic analysis, and the absence of experimental validation to identify specific glucose metabolism–related factors associated with childhood asthma. We recruited age‐matched children with newly diagnosed allergic asthma without chronic inhaled corticosteroid use, yet BMI, sex, feeding patterns, pet contact, secondhand smoke exposure, geographic location, and asthma severity were not controlled. This methodological limitation leads to unavoidable confounding bias. Larger, well‐matched case–control cohorts are needed in follow‐up independent studies to verify our preliminary results. Finally, due to the absence of individual‐level data, this study cannot thoroughly investigate more complex relationships and may overlook the nonlinear associations among gut microbiota, immune cell phenotypes, and childhood asthma. Although MR analysis and metagenomic sequencing can estimate causal relationships between exposure factors and disease outcomes to a certain extent, the conclusions drawn from this study still need further verification based on additional basic experiments and clinical research.

## 5 Conclusion

In summary, we identified 7 types of gut microbiotas and 25 types of immune cells that are causally related to childhood asthma. We identified 2 mediating relationships, including 2 immune cell–mediated causal pathways from gut microbiota to childhood asthma. In addition, we employed metagenomic sequencing for the validation of MR analysis results using clinical samples. Recent findings highlight the mediating role of immune cell traits between gut microbiota and childhood asthma, opening up new avenues for future research. Longitudinal studies will provide insight into the evolving interactions between gut microbiota, immune cell traits, and childhood asthma progression, distinguishing causal associations and correlations. Furthermore, validating these associations through functional assays in animal models and exploring intervention strategies could lay the foundation for new therapeutic approaches. In essence, revealing the complex relationship between specific gut microbiota exposures, their mediators, and childhood asthma underscores the need for comprehensive studies to leverage these insights for clinical advancement.

## Author Contributions

All authors have made significant contributions to the reported work. Rui Shi and Zongming Yang were involved in conception, study design, execution, data acquisition, analysis, and manuscript writing. Xiuyun Zhou, Dong Xu, Wankai Xue, Lanfen An, and Xiaole Zhang have reviewed the manuscript. Yongjian Huang was involved in the conception of the study and critical review of the article. All authors have provided final approval of the version to be published, agreed on the journal to which the article will be submitted, and agreed to take responsibility for all aspects of the work.

## Funding

The study was supported by the National Natural Science Foundation of China (82403847) and Special Fund for Nursing Research of Tongji Hospital (2023D43).

## Ethics Statement

This study was approved by the Ethics Committee of Tongji Hospital, Tongji Medical College, Huazhong University of Science and Technology (Approval No.: TJ‐IRB202512024). Written informed consent was obtained from the legal guardians of all participating children in accordance with the Declaration of Helsinki. All clinical samples and relevant clinical data were collected and used in strict compliance with the approved ethical protocols, and all personal identifying information of the participants was anonymized to ensure confidentiality.

## Consent

The authors have nothing to report.

## Conflicts of Interest

The authors declare no conflicts of interest.

## Supporting information


**Figure S1:** The heterogeneity, multiplicity and sensitivity tests of the MR analysis between gut microbiotas and childhood asthma.

**Figure S2:** The leave‐one‐out sensitivity analysis of the MR analysis of eight genera on childhood asthma.

**Figure S3:** The leave‐one‐out sensitivity analysis of the MR analysis of 25 immunophenotypes and childhood asthma.

**Figure S4:** The leave‐one‐out sensitivity analysis of the MR analysis of 25 immunophenotypes and childhood asthma.

**Figure S5:** The heterogeneity tests, multiplicity tests, sensitivity tests, and the leave‐one‐out sensitivity analysis of the MR analysis between gut microbiotas and immunophenotypes.

**Figure S6:** KEGG enrichment pathways and virulence factors in the asthma group and healthy control group. (A) KEGG enrichment pathways between the two groups identified via LEfSe analysis. (B) Heatmap of differential virulence genes between the two groups (top 20) identified via LEfSe analysis. (C) Bar chart of the top 10 virulence genes. *p < 0.05, **p < 0.01.

**Table S1:** The *F*‐statistics ranged from 20 to 60.93.

**Table S2:** The 52 SNPs for these seven gut microbiota types.

**Table S3:** The reverse MR analysis between gut microbiota and childhood asthma.

**Table S4:** The Steiger filtering of gut microbiota and childhood asthma.

**Table S5:** The SNP of 731 immunophenotypes.

**Table S6:** Heterogeneity and multiplicity tests of the relationship between these immunophenotypes and childhood asthma.

## Data Availability

The datasets used and/or analyzed during the current study are available from the corresponding author on reasonable request.
